# Supplementary material for: Screening for the 137Cs body burden owing to the Chernobyl accident in Zhytomyr region, Ukraine: 2009–2018
Source: PLoS One. 2021 Jan 15;16(1):e0245491. doi: 10.1371/journal.pone.0245491 (PMC7810316; doi:10.1371/journal.pone.0245491)
Supplement: Attachment — Submitted filename: Response to reviewers.doc [file pone.0245491.s001.doc]

December 11, 2020

Dear Reviewers,

Thank you for inviting us to submit a revised draft of our manuscript entitled, ‘Screening for the ^137^Cs Body Burden due to the Chernobyl Accident in Korosten city, Zhytomir, Ukraine: 2009–2018’ [ms. no PONE-D-20-16627] to *PLoS ONE*. We also appreciate the time and effort you and each of the reviewers have dedicated to providing insightful feedback on ways to strengthen our paper. Thus, it is with great pleasure that we resubmit our article for further consideration. We have incorporated the changes that reflect the detailed suggestions you have so graciously provided. We also hope that our edits and the responses we provide below satisfactorily address all the issues and concerns you and the reviewers have noted.

We would like to bring to your attention the fact that we have revised the title of our manuscript to “**Screening for the ^137^Cs Body Burden owing to the Chernobyl Accident in Zhytomyr region, Ukraine: 2009–2018**” with the aim of improving the clarity of the research area. Also, we had native speakers of English proofread our English writing again.

To facilitate your review of our revisions, the following is a point-by-point response to the questions and comments delivered in your letter dated November 16, 2020.

- **Reviewer #1:**

We are grateful to reviewer #1 for the critical comments and useful suggestions that have helped us to improve our paper considerably. As indicated in the responses that follow, we have taken all these comments and suggestions into account in the revised version of our paper.

**Comment 1.** ‘In abstract line no. 22, replace radioactive materials by fission products’.

**Response.**

We have edited the sentence in accordance with the suggestion made by reviewer #1.

(P2, L22)

**Comment 2.** ‘Reference should be revised as per PLOS ONE journal style’.

**Response.**

We have checked the references and ensured that they comply with the formatting guidelines of PLOS ONE.

- **Reviewer #2:**

We are grateful to reviewer #2 for the critical comments and useful suggestions that have helped us to improve our paper considerably. As indicated in the responses that follow, we have taken all these comments and suggestions into account in the revised version of our paper.

**Comment 1.** ‘Line 69-70: "...received annual effective doses of less than 1 mSv in ^137^Cs addition to the natural background doses of from the Chernobyl fallout." This reads as if Chernobyl fallout were natural background.’.

**Response.**

We have rewritten the sentence to ensure that it conveys the accurate meaning and provides easier comprehension. (P5, L69–72)

**Comment 2.** ‘Line 74-75: "...reported high frequency of people with an estimated internal effective dose...” This required editing; zero can be an estimated dose.’

**Response.**

We have incorporated a phrase that is widely used in other similar studies. We hope it clarifies the implication of the sentence. (P5, L75-77)

**Comment 3.** ‘Line 77: "...the effective dose of internal exposure is becoming increasingly important…” This phraseology is misleading. The fraction of the dose from internal exposure is becoming larger, but it is a lager fraction of decreasing dose.’

**Response.**

We have modified the sentence to provide more clarity and have also included the additional reference to another study that drew the similar conclusion. (P5, L78–81)

**Comment 4.** ‘Line 104-106: The concept of “contamination level zones” required some introduction. Simply referring to smaller digits is not clear. Add a sentence of two to describe the classification of “levels of contamination”.’

**Response.**

We have added the description of the contamination level zones and ranges of amount of ^137^Cs deposited in each corresponding zone in the Materials and Methods section. (P7, L102–117)

**Comment 5.** ‘Line 116-119: It is not clear if this is a font change or if the small print of the caption for Figure 1. The concepts of “contamination levels” is not clear (and are not illustrated in Figure 1.)’.

**Response.**

We have removed Figure 1 and its caption from our manuscript owing to issues related with obtaining permission from the copyright holder.

**Comment 6.** ‘Line 124: Does the participant lean his abdomen on the seat?’.

**Response.**

We have edited and added clarification to the process of placing the abdomen. (P8, L126–127)

**Comment 7.** ‘Line 140: This “does coefficient” requires a reference. Does it assume uniform distribution of Cs in the body? The term “dose coefficient” in the health physics literature has a different meaning (usually Sv per Bq intake). What are the underlying assumptions? – continuous intake and contamination at that level? For what period – one year.’

**Response.**

We have revised the sentence with and included a reference about the “dose coefficient” calculation. (P8, L141–143)

**Comment 8.** ‘Line 148 et seq.: How were means calculated? A very large fraction of measurements are below detection (the median is always less than detectable); were they assigned zero? A minimum level? A log-extrapolated mean?’

**Response.**

We have included an additional sentence describing how the values of those whose internal exposure was below detectable level were classified as having “0 Bq”. (P9, L151–153)

**Comment 9.** ‘Line 177-178 and similar places below: “The 75^th^ percentile was detected only for 3 years in the earlier part of the study,” Awkward wording. Perhaps “More than 25 percent of measurements exceeded the detection level only for 3 years…”’

**Response.**

We have revised and extended the description of the 75^th^ percentile to reflect your comments. (P12, L182–184)

**Comment 10.** ‘Line 185-186: Same comments as above. “More than 25% of the measurements exceeded the minimum detectable level in 2009, 2011, and 2012…”’

**Response.**

We have revised and extended the description of the 75^th^ percentile to reflect your comments. (P12, L190–192)

**Comment 11.** ‘Line 206-207: “all belonging to various areas.” Awkward wording. Perhaps “…from various locations in the study domain.”’

**Response.**

This has been amended according to the comment. (P13, L213)

**Comment 12.** ‘Line 228 and often hereafter: “detectable people”. Change to “people with detectable contamination”. Also line 236, 240, 243, 244, Table 4, 251-251, 257, 262, 389, 390, 430, 433-434.’

**Response.**

We have replaced “detectable people” by the term recommended in the comment to all the applicable parts of the manuscript.

**Comment 13.** ‘Line 231: Discussions of statistical significance in this paper are difficult to follow – I suspect that the translator was not familiar with the concept. Here, what does “were insignificant” refer to? Similarly Line 272, 358.’

**Response.**

We have improved all the references made to significance and added a more explicit description. (P15–16, L233–240)

**Comment 14.** ‘Line 254: “...average internal Bq/kg body burden…” This is poor wording. Perhaps “average Cs tissue concentrations (Bq/kg)…” or something like that.’

**Response.**

We have amended the wording to reflect the meaning of your comments. (P17, L260)

**Comment 15.** ‘Line 285-286: “The 75^th^ percentile for Bq/kg was identified in only 3 different years at the beginning part of the study.” Again, there was always a 75^th^ percentile – it was at a level below detection. Awkward wording.’

**Response.**

We have revised and extended the description of the 75^th^ percentile to reflect your comments. (P20, L293–295)

**Comment 16.** ‘Line 295: “…and showed significance.” Perhaps “…with statistical significance.”’

**Response.**

We have rewritten the sentence above by incorporating details mentioned in your comment. (P20, L301–303)

**Comment 17.** ‘Line 308-309: ICRP does not set regulatory limits, only generic recommendations. The UA government sets the applicable regulations… what are they?’

**Response.**

We have revised the sentence in accordance with the comments. (P21, L316–317)

**Comment 18.** ‘Line 362-363: What does significance imply here?’

**Response.**

We elaborated on this section with a more explicit description. (P23, L363–371)

**Comment 19.** ‘Line 367-368: “…cesium and socio-economic conditions…” this reads as if socioeconomic conditions were continuously decaying…’

**Response.**

We have revised the section and improved on the poor wording. (P24, L376–378)

**Comment 20.** ‘Line 379-380: forest products are also “locally produced” (perhaps more so than store-bought foods!).’

**Response.**

We have replaced the above-mentioned term with the term “kitchen-garden products”. (P24, L388–389)

**Comment 21.** ‘Line 394-395: “The average Cs 137 concentration in men exceeded THAT IN WOMEN by a factor of 1.6”.’

**Response.**

We have revised the sentence in accordance with the comment. (P25, L403–404)

**Comment 22.** ‘Line 399: “The reasons for this difference include slower excretion of Cs 137 from the male body and POTENTIALLY larger quantities of consumption…” You are making an assumption here about the reason – you did not measure it or ask dietary questionnaires. Similar comment in other nearby locations where you are speculating on the reasons.’

**Response.**

We have revised the sentence in accordance with the comment. (P25, L408–410)

**Comment 23.** ‘Line 441: individuals do not “exhibit radioactivity”.’

**Response.**

We have revised the sentence in accordance with the comment. (P27, L448–454)

**Comment 24.** ‘Line 447: “…which is much shorter…” Perhaps “which is short in relation to the counting intervals”.’

**Response.**

We have revised the sentence in accordance with the comment. (P27, L457–459)

**Comment 25.** ‘Line 451: “ununiform” should be nonuniform. Also, on line 453.’

**Response.**

We have revised the sentence in accordance with the comment. (P27, L461–462)

With the incorporation of these amendments to our final manuscript, we hereby resubmit our manuscript for a secondary evaluation. We hope that the revised version of our paper is now suitable for publication in *PLoS ONE*, and we look forward to hearing from you at your earliest convenience.

Sincerely,

Naomi Hayashida, M.D., PhD.

Professor of Division of Strategic Collaborative Research, Center for Promotion of Collaborative Research on Radiation and Environment Health Effects, Atomic Bomb Disease Institute, Nagasaki University

1-12-4 Sakamoto, Nagasaki 852-8523, JAPAN

TEL: +81-95-819-8507

FAX: +81-95-819-8508

E-mail: [naomin@nagasaki-u.ac.jp](mailto:naomin@nagasaki-u.ac.jp)
